# Supplementary material for: The interactome of intact mitochondria by cross-linking mass spectrometry provides evidence for coexisting respiratory supercomplexes
Source: Mol Cell Proteomics. 2017 Dec 8;17(2):216–32. doi: 10.1074/mcp.RA117.000470 (PMC5795388; doi:10.1074/mcp.RA117.000470)
Supplement: Supplemental Data [file supp_RA117.000470_133922_0_supp_23978_fzffwf.zip › spectra_annotation/mito_DR_spectra_annotation/29-1-13-1-19-1.pdf]

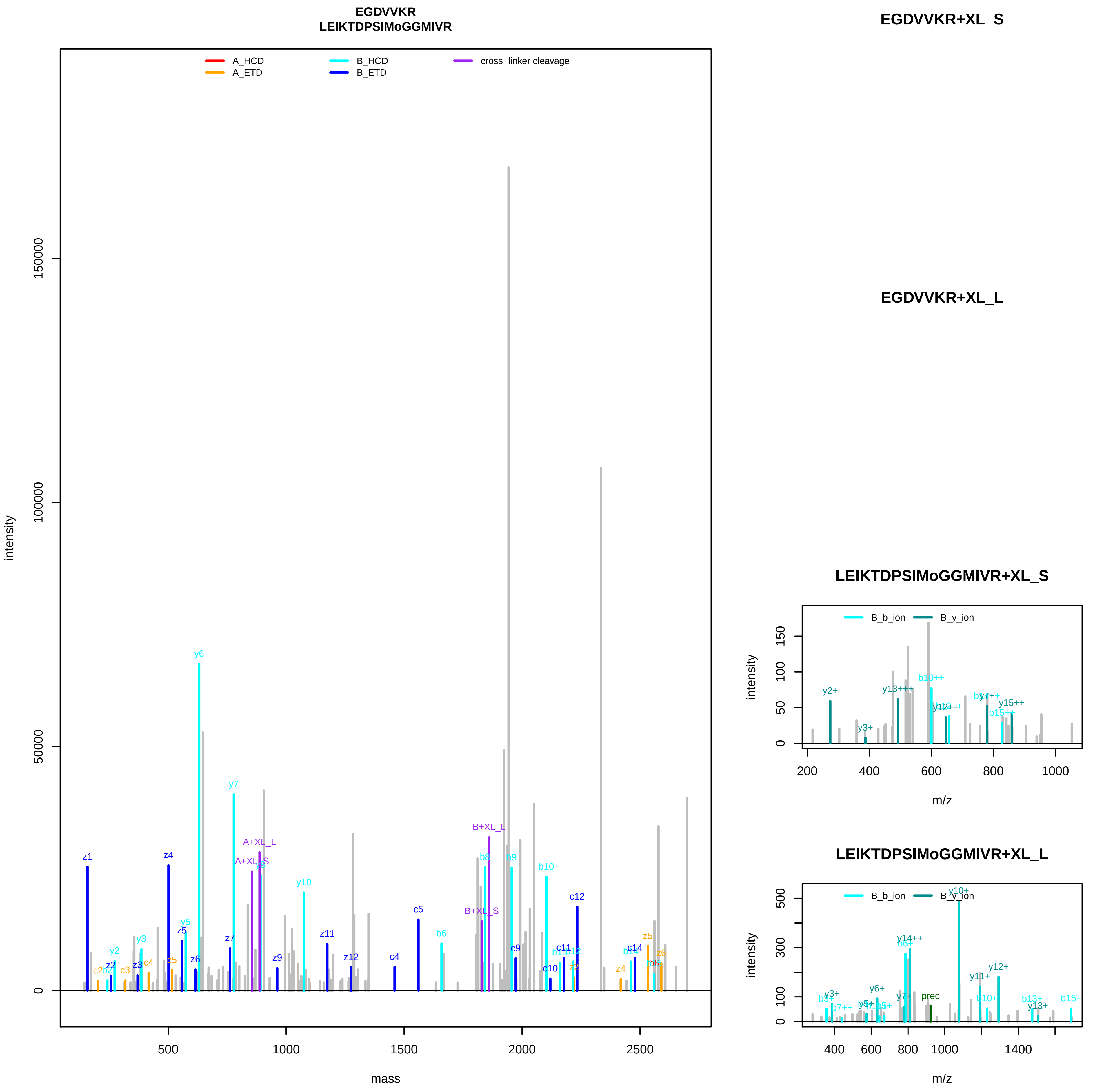
EGDVVKR
LEIKTDPSIMoGGMIVR
A_HCD
A_ETD
B_HCD
B_ETD
cross−linker cleavage
intensity
mass
0
50000
100000
150000
500
1000
1500
2000
2500
z1
z4
y6
y7
A+XL_L
A+XL_S
y10
c5
B+XL_L
b8
b9
b10
c12
B+XL_S
y5
z5
y3
y2
z2
z3
c4
c5
z6
z7
z9
z11
z12
c4
b6
c9
c10
c11
b12
z2
z4
c14
b14
z5
z6
b6
EGDVVKR+XL_S
EGDVVKR+XL_L
LEIKTDPSIMoGGMIVR+XL_S
B_b_ion
B_y_ion
intensity
m/z
0
50
100
150
200
400
600
800
1000
y2+
y3+
y13+++
b10++
y12++
y7+
y15++
b15++
LEIKTDPSIMoGGMIVR+XL_L
B_b_ion
B_y_ion
intensity
m/z
0
100
300
500
400
600
800
1000
1400
y10+
y14++
b6+
y6+
y3+
b3+
b7++
y5+
y7+
prec
y11+
y12+
b10+
b13+
y13+
b15+
